# Extracorporeal Shock Wave Therapy for Lateral Epicondylitis, Lonely or in Combination with Topical Corticosteroid; Which Approach is Superior?

**DOI:** 10.31661/gmj.v9i0.1791

**Published:** 2020-07-01

**Authors:** Babak Vahdatpour, Parisa Taheri, Fatemeh Abasi

**Affiliations:** ^1^Department of Physical Therapy and Rehabilitation, Isfahan University of Medical Sciences, Isfahan, Iran

**Keywords:** Extracorporeal Shockwave, Lateral Epicondylitis, Transdermal Drug Delivery

## Abstract

**Background:**

Lateral epicondylitis (LE) is a common musculoskeletal disorder. Although varieties of modalities have been proposed for its treatment, the outcomes are uncertain, and the responses would diminish early by the time passage. The current study was aimed to assess the efficacy of extracorporeal shock wave therapy (ESWT) merely and in combination with topical corticosteroid for the treatment of LE.

**Materials and Methods:**

In the current double-blinded randomized clinical trial, 70 patients with the diagnosis of LE were randomly allocated to two intervention groups of ESWT merely (control group) (n=35) or ESWT plus topical corticosteroid (intervention group) (n=35). The ESWT was performed weekly for three weeks. Topical clobetasol was utilized within 30 minutes before ESWT for the intervention group, while Vaseline gel was used in a similar pattern for controls. Pain based on a visual analog scale (VAS), handgrip strength (HGS) and the Patient-Rated Tennis Elbow Evaluation (PRTEE) were assessed for the patients before the intervention, following the intervention cessation, and within two months post-intervention.

**Results:**

Statistically significant improvement was found following both interventions in terms of pain, HGS, and function (P-value<0.001 for all), while the comparison of the two interventions, ESWT, merely versus in combination with topical clobetasol, revealed insignificant difference (P-value>0.05).

**Conclusion:**

The findings of our study are in favor of ESWT use either merely or in combination with topical steroids for the treatment of LE, while the comparison of the two techniques revealed insignificant differences.

## Introduction

Lateral epicondylitis (LE) or tennis elbow syndrome is a common upper-extremity musculoskeletal disorder associated with tenderness over the lateral epicondyle of the humerus [1]. This syndrome that is mostly presented in the 3rd and fourth decades of life affects 1–3% of the adult population without significant gender predominance [2]. The tendinous origin of the extensor carpi radials brevis muscle is the primary area of pathologic changes responsible for the LE presentations. The pathologic changes occur due to overuse or repetitive trauma in this area leading to fibrosis and microtears in the involved tissues. Therefore, resisted extension of the wrist and fingers, and the forearm supination causes remarkable pain sensation, remarkably disturb the upper extremity functioning [2,3]. The keystone goals of LE treatment are to reduce pain, preserve the motion, flexibility, and strength of the upper extremity, and improve the endurance of non-operative management of this syndrome. Studies in the literature have represented up to 95% of the success rate for the non-operative management of LE. Physical therapy, activity modification, non-steroidal anti-inflammatory drugs (NSAIDs), and corticosteroid injections are the popular non-operative modalities used for the treatment of LE [3,4]. Extracorporeal shock wave therapy (ESWT) is another approach approved by the Food and Drug Association (FDA) for the treatment of this syndrome. This approach, known as Radial Shock Wave Therapy (RSWT), has been raised for a short time [5,6]. RSWT is the approach favored for the cases representing refractory lateral epicondylitis. This approach is usually applied to the injured tissue to help revascularization and stimulate or reactivate the process of connective tissue and bone healing, thereby relieving pain and improving function [7]. There are conflicting reports in the literature regarding the effectiveness of RSWT. Some of the studies have represented a significant improvement in handgrip strength (HGS), pain score, and functional score after RSWT [8], while others have only represented a placebo effect for this approach of treatment [9]. There are studies in the literature shown that techniques such as Iontophoresis and phonophoresis can promote drug absorption through the skin at the site of injury [10-12]. Therefore, we have raised the hypothesis that extracorporeal shock wave therapy may facilitate the delivery of topical corticosteroid to the deep layers beyond the affected lateral epicondyle. The current study aims to assess the efficacy of topical corticosteroid use in combination with ESWT on the pain and handgrip strength (HGS) of patients with the presentations of lateral epicondylitis.

## Materials and Methods

###  Study Population

The current report is a double-blinded randomized clinical trial (RCT) conducted on 70 patients with the clinical diagnosis of lateral epicondylitis referred to the Physical Medicine and Rehabilitation Clinic of Alzahra Hospital affiliated at Isfahan University of Medical Sciences from September 2018 to May 2019. The patients who referred with chronic lateral epicondylitis for over three months who had the least age of 18 years old and were irresponsible to non-invasive conservative approaches (including non-steroidal anti-inflammatory drugs, and physiotherapy plus phonophoresis) were included. Patients’ reluctance for fulfilling the study protocol, pregnancy during the treatment, diagnosis of malignancy or rheumatologic inflammatory diseases, generalized polyarthritis, concurrent shoulder dysfunction in the affected side of LE, and elbow arthritis, and requirement of surgical procedure on the affected elbow, and suspecting radial tunnel syndrome based on the clinical presentations and physical examinations [13] were considered as the exclusion criteria from the study. The Ethics Committee of Isfahan University of Medical Sciences confirmed the study protocol primarily. After that, the study design was explained for the patients entirely, and written consent for participation in the study was obtained. The study population was randomly allocated to two approaches, including ESWL plus topical corticosteroid (n=35) (intervention group) or mere ESWL (n=35) (control group). The randomization was done using Random Allocation software (GraphPad software, Inc., California, USA), in which each of the patients was provided with a particular number, allocated her/him to one of the groups. The patients, the operator of ESWL, and the person who analyzed the function of affected hands were blinded to the type of intervention, as neither the patients nor the analyzer was aware of the code numbers used for clobetasol tube and Vaseline oils.

###  Diagnosis and Management

 The diagnosis of LE was initially made by a physical examination done by a skilled target physical medicine and rehabilitation specialist; therefore, the sensation of tenderness on the location of the extensor tendons of the lateral epicondyles was considered as LE. Then, the diagnosis was confirmed if the relevant tests of Thomsen and Maudsley were positive.

 The intervention and control groups received routine lateral epicondylitis ESWL treatment as follows; weekly ESWT intervention for three sessions using Radial-ESWT (Storz Medical Duolith SD1 machine) with a protocol of 2000 shockwaves, 1.2 – 2.2 mJ/mm2 of energy and frequency of 5 Hz. In the intervention group, patients also received topical corticosteroids. To reduce bias and preserving blindness, topical clobetasol ointment and Vaseline oil similar in shape, color, and tube were coded. The intervention group received code one ointment tubes containing clobetasol within 30 minutes before ESWT, and the latter group received tubes coded 2 containing Vaseline oil with a similar pattern. Conventional modalities for the treatment of lateral epicondylitis including splint (forearm band during the day, mostly during activity, until the end of the shockwave period) and exercise (stretching the elbow extensor muscles three times daily for 15 seconds) were prescribed for both of the intervention and control groups.

###  Primary Outcomes

 Sociodemographic characteristics of the patients, including age, gender, and symptoms onset, were recorded in the study checklist.

 Other investigations of the current study included handgrip strength, pain, and the Patient-Rated Tennis Elbow Evaluation (PRTEE) questionnaire. The assessments were done before the interventions, immediately after the cessation of the last session of interventions, and eventually, within two months following the interventions. A target expert specialist performed all of the evaluations.

###  Handgrip Strength

Handgrip strength was evaluated using a Jamar Hydraulic Hand Dynamometer (Sammons Preston Rolyan, Bolingbrook, IL). The patients were requested to squeeze the hand dynamometer as much as tolerable for them (the maximum strength they could make) in standard position as they were sitting, their elbow was 90 degrees flexed, the shoulder was adducted and neutrally rotated, the wrist was slightly extended and ulnar deviated, and the forearm was neutrally positioned [14]. This test was done three times with a 15-second interval.

###  Pain Assessment

 The pain assessment o the patients was performed using the Visual Analogue Scale, a means that rates from 0-10 in which zero equals no pain, and by an increase in pain, the score increases to 10 as the most severe pain sensation. The pain scores are assessed twice, at rest, and post-wrist activity. Then the average of the scores is considered as the pain score for each time of the assessments.

###  Patient-Rated Tennis Elbow Evaluation

The Patient-Rated Tennis Elbow Evaluation (PRTEE) is a means to individually assess the pain and functioning of the affected elbow with lateral epicondylitis. This means of measurement is composed of two entities, including pain and function subscales. The pain is assessed at rest and during activities involving the elbow. The function subscale assesses the difficulty of specific and usual activities within the past week. The scores are evaluated separately and all together, scoring from 0 to 100 [15].

###  Statistical Analysis

 The obtained data were entered into the Statistical Package for Social Sciences (SPSS) version 23 (SPSS Inc., Chicago, IL, USA). The descriptive data were presented in mean, standard deviation, percentages, and absolute numbers. For analytics, independent t-test, Mann Whitney test, repeated measure, and Bonferroni test for post hoc test were used. P-value of less than 0.05 was considered as a significant level.

## Results

 The current study has been conducted on 76 patients with the chief complaint of lateral epicondylitis, among which 70 ones fulfilled the study protocol, and the remained six ones did not refer for the follow-up visits. The study population was divided into two subgroups of treatment with ESWT merely or in combination with topical corticosteroids. The two assessed groups were similar in terms of age, gender distribution, and duration of LE onset (P-value>0.05). Detailed demographic data of the studied population is demonstrated in Table-1. Table-2 represents the primary outcomes measured in the current study. Based on this table, the use of ESWT merely or in combination with topical clobetasol led to significant improvement in pain, HGS, and PRTEE scores (P-value<0.05). Besides, the participants presented improved outcomes within the time (P-value<0.05), as well. The general comparison of the two interventions with each other showed insignificant differences between two modalities in terms of HGS (P-value=0.95) and PRTEE in all entities including pain (P-value=0.36), function (P-value=0.60) and total subscales (P-value=0.82), while the pain assessment using VAS found a significant superiority of combination therapy to the mere use of ESWT (P-value=0.005).

## Discussion

To the best of our knowledge, the current study is the first one assessing the combination use of topical corticosteroid plus ESWT for the treatment of lateral epicondylitis. We divided the patients into two groups of intervention with ESWT merely or in combination with topical clobetasol. The two groups were similar in demographics; therefore, the probable confounding role of demographics on the outcomes has been eliminated. In this report, we found a dramatic response of the patients to ESWT therapy, whether merely or in combination with topical clobetasol, while the comparison of the two interventions revealed insignificant differences between the two assessed groups except for pain analysis using VAS. As previously stated, varieties of modalities have been introduced in order to improve the topical absorption of the drugs. Iontophoresis and phonophoresis are the two most popular ones emerging to improve the cutaneous drug penetration through stratum corneum; the subcutaneous tissue principally acts as a barrier against the percutaneous absorption of the agents [11,16]. On the other hand, studies in the literature have demonstrated that shock waves can facilitate the transmission of macromolecules into the deep dermis by emerging disruptions in the hydrophilic domains lying on the stratum corneum [17,18]. This transient increase in the permeability of dermis may substantially occur due to the generation of cavitations bubbles. The generation of these cavitations not only does not cause cell death but also provides the condition to easily transfer the large molecules into the cells [19,20]. Furthermore, studies are showing the capability of ultrasound energy to make cavitational effects that, in response, can limit the efficacy of skin barrier [21]. In this order, we designed the current study to enhance topical corticosteroid analgesic effect by the concurrent use of ESWT. Several studies in the literature have assessed the use of ESWT for the treatment of LE with uncertain responses to the treatment. Mehra *et al*. were among the first groups assessing ESWT for the treatment of EL. They followed their study population for six months, and the primary outcome of this study was pain improvement only. Their conclusion was in favor of ESWT use as they represented 78% of remarkable pain relief in their patients [22]. Rompe *et al*. were the other group of scientists who assessed the use of low-energy ESWT and followed their patients for a year. They found a considerably higher rate of patients capable of properly perform activities at desired levels. Besides, the pain relief was more prominent in ESWT-treated patients than placebo [23]. Similar outcomes versus placebo were represented in other studies as well [24-26]. Besides to placebo-controlled trials, other studies are expressing their outcomes in terms of ESWT use versus other techniques. Wong *et al*. conducted their study in terms of assessing the efficacy of ESWT with a similar pattern in our study versus acupuncture therapy twice weekly for three weeks. They eventually represented a significant reduction in the pain sensation by both of the approaches, while the HGS did not considerably improve within two weeks of follow-ups, although the gradual improvement was found in both interventions. A comparison of the two approaches showed insignificant difference [27]. On the other hand, Vulpiani *et al*. conducted a 12-month follow-up study in order to compare the efficacy of EWST versus cryoultrasound therapy. They followed their patients twice within 6 and 12 months and represented that although both of the approaches led to improved pain, HGS, and function, ESWT was remarkably superior to cryoultrasound therapy [28]. Lee *et al*. were the other groups of scientists who compared the efficacy of ESWT versus local steroid injection, and despite the remarkable improvement in all entities of pain, HGS and function within 4 and 8 weeks of follow-up for both of the modalities, they stated superior outcomes of ESWT [29]. Similar results were presented by Gündüz *et al*., who compared local steroid injection versus physical therapy and ESWT. They followed their patients for a year and represented the dramatic response of all modalities in short-term assessments, while the improved outcomes remained for a year only in the ESWT treated patients [30]. Contrary to our findings and studies above, there are even studies representing the inefficiency of ESWT for the treatment of LE. Studies performed by Capan *et al*. [14], Staples *et al*. [31], and Buchbinder *et al*. [32], who have unanimously supported the idea that ESWT may not be better than placebo or only a little evidence is available about the beneficence of this approach than placebo. We think these outcomes may have achieved due to the number of studied population or due to the course of follow-ups or to a more extent due to the pattern of ESWT performance regarding the numbers of sessions and the energy used for the performance of this approach. In summary, respectful to most of the studies in the literature, we found a significant response to ESWT therapy for tennis elbow syndrome. Still, as the first study assessing the use of topical corticosteroids in combination with ESWT, however, the patients represented dramatic response in all entities of pain, HGS, and function, we found no remarkable differences between mere use of ESWT versus in combination with topical clobetasol.

## Limitations

 One of the significant limitations of our study is not to match the parameters, including job, limb dominancy, involved side, and pretreatment used medications. Besides, in the current report, we have followed the patients for a short time. Therefore, as the mentioned factors may influence the outcomes, we recommend further studies by matching the parameters and longer duration of follow-up.

## Conclusion

 The findings of our study are in favor of ESWT use either merely or in combination with topical steroids for the treatment of LE, while the comparison of the two techniques revealed insignificant differences.

## Acknowledgment

 We want to acknowledge Dr. Safaei, who helped us in the preparation and edition of the current paper.

## Conflict of Interest

 Authors of the current report, present no conflict of interest.

## Financial Support

 Isfahan University of Medical Sciences sponsored the study (Grant number: 397452).

**Table 1:** Demographic Information of the Studied Population

	**Intervention Group** **( n =35)**	**Control Group ** **(n=35)**	**P-value**
**Age ( mean ± SD ) ** **(years)**	41.5±10.2	39.4±9.3	0.46
**Gender (F/M) (%)**	19 (54.3%)/ 16 (45.7%)	20 (57.14%)/ 15 (42.8%)	0.64
**Disease duration ( mean ± SD ) ** **(months)**	7.02±2.62	7.652.87	0.66

**Table 2:** Comparison of Pain Score, Handgrip Strength, and Patient-Rated Tennis Elbow Evaluation between Intervention Versus control Groups

	**Intervention Group** **(n=35)** **( mean±standard deviation)**	**Control Group** **(n=35)** **( mean±standard deviation)**	**P1**	**P2** **(Intervention)**	**P3** **(Time)**	**P4** (Intervention*Time)
**Pain (visual analogue scale)**
**Before the intervention**	8.14±.98	8.17±.89	0.96	<0.001	<0.001	0.005
**Immediately after intervention cessation**	4.65±1.23	5.65±1.23	0.001			
**Within two months after the intervention**	4.54±1.65	5.65±1.16	<0.001			
**Handgrip strength**
**Before the intervention**	17.94±3.68	19.04±3.49	0.10	<0.001	<0.001	0.95
**Immediately after intervention cessation**	21.14±4.39	21.14±3.23	0.74			
**Within two months after the intervention**	21.94±4.35	21.54±2.72	0.58			
**Patient-Rated Tennis Elbow Evaluation (pain subscale)**
**Before the intervention**	40.40±4.25	38.20±4.31	0.03	<0.001	<0.001	0.36
**Immediately after intervention cessation**	33.00±4.61	33.02±4.18	0.92			
**Within two months after the intervention**	32.65±4.67	32.11±4.20	0.68			
**Patient-Rated Tennis Elbow Evaluation (function subscale)**
**Before the intervention**	36.94±4.10	34.94±4.51	0.03	<0.001	<0.001	0.60
**Immediately after intervention cessation**	30.45±3.86	32.22±3.91	0.21			
**Within two months after the intervention**	30.05±3.97	31.74±3.77	0.17			
**Patient-Rated Tennis Elbow Evaluation (total)**
**Before the intervention**	77.20±8.24	72.85±8.56	0.34	<0.001	<0.001	0.82
**Immediately after intervention cessation**	63.54±7.54	65.25±7.66	0.39			
**Within two months after the intervention**	62.71±7.81	64.14±7.69	0.49			

**P1 :** Mann-Whitney test, **P2:** GLM
